# Supplementary material for: Improved genetic prediction of complex traits from individual-level data or summary statistics
Source: Nat Commun. 2021 Jul 7;12:4192. doi: 10.1038/s41467-021-24485-y (PMC8263809; doi:10.1038/s41467-021-24485-y)
Supplement: Supplementary file 3 — Description of Additional Supplementary Files [file 41467_2021_24485_MOESM3_ESM.pdf]

Description of Additional Supplementary Files

File Name: Supplementary Data 1
Description: Supplementary Data 1: Details, SNP heritabilities and prediction accuracies for all 225 UK Biobank phenotypes. For each phenotype, we report the UK Biobank data field ID (irnt indicates the phenotype was inverse-rank-normaltransformed), the total number of samples and, for binary traits, the numbers of controls and cases. We also report estimates of SNP heritability (computed using SumHer assuming the GCTA, LDAK-Thin or BLD-LDAK Models), and estimates of R 2 , the squared correlation between observed and predicted phenotypes, for prediction models constructed using LDAK-Lasso-SS, LDAKRidge-SS, LDAK-Bolt-SS and LDAK-Bayes-SS (assuming the GCTA, LDAK-Thin or BLD-LDAK Models). The 14 phenotypes in bold are those for which we also have individual-level data. The final column reports the best-performing tool and heritability model for each phenotype. Note that LDAK-Lasso-SS, LDAKRidge-SS, LDAK-Bolt-SS and LDAK-Bayes-SS, when run assuming the GCTA Model, represent our implementations of lassosum, sBLUP, LDpred and SBayesR, respectively.
