# Supplementary material for: Improved genetic prediction of complex traits from individual-level data or summary statistics
Source: Nat Commun. 2021 Jul 7;12:4192. doi: 10.1038/s41467-021-24485-y (PMC8263809; doi:10.1038/s41467-021-24485-y)
Supplement: Supplementary file 5 — Reporting Summary [file 41467_2021_24485_MOESM5_ESM.pdf]

nature research

Corresponding author(s): Doug Speed

Last updated by author(s): May 17, 2021

Reporting Summary

Nature Research wishes to improve the reproducibility of the work that we publish. This form provides structure for consistency and transparency in reporting. For further information on Nature Research policies, see our Editorial Policies and the Editorial Policy Checklist.

Statistics

For all statistical analyses, confirm that the following items are present in the figure legend, table legend, main text, or Methods section.

n/a	Confirmed	
☐	☒	The exact sample size (n) for each experimental group/condition, given as a discrete number and unit of measurement
☐	☒	A statement on whether measurements were taken from distinct samples or whether the same sample was measured repeatedly
☐	☒	The statistical test(s) used AND whether they are one- or two-sided<br>*Only common tests should be described solely by name; describe more complex techniques in the Methods section.*
☐	☒	A description of all covariates tested
☐	☒	A description of any assumptions or corrections, such as tests of normality and adjustment for multiple comparisons
☐	☒	A full description of the statistical parameters including central tendency (e.g. means) or other basic estimates (e.g. regression coefficient) AND variation (e.g. standard deviation) or associated estimates of uncertainty (e.g. confidence intervals)
☐	☒	For null hypothesis testing, the test statistic (e.g. F, t, r) with confidence intervals, effect sizes, degrees of freedom and P value noted<br>*Give P values as exact values whenever suitable.*
☐	☒	For Bayesian analysis, information on the choice of priors and Markov chain Monte Carlo settings
☒	☐	For hierarchical and complex designs, identification of the appropriate level for tests and full reporting of outcomes
☐	☒	Estimates of effect sizes (e.g. Cohen's d, Pearson's r), indicating how they were calculated

Our web collection on statistics for biologists contains articles on many of the points above.

Software and code

Policy information about availability of computer code

Data collection	No software was used for data collection (we only used previously-collected data - primarily from UK Biobank).
Data analysis	In total, we used eight new tools and ten existing tools. Seven of the new tools (LDAK-Ridge-Predict, LDAK-Bolt-Predict, LDAK-BayesR-Predict, LDAK-Lasso-SS, LDAK-Ridge-SS, LDAK-Bolt-SS 41and LDAK-BayesR-SS) are contained within our software LDAK (we used version 5.1). The remaining new tool (big_spLinReg) is contained within our software bigstatsr (we used version 1.5.1). For the ten existing tools, we used seven software. For Lasso we used bigstatsr (version 1.5.1). For BLUP and Classical PRS, we used LDAK (version 5.1). For Bolt-LMM, we used the software of the same name (version 2.3.5). For BayesR, SBLUP and SBayesR, we used gctb (version 2.0). For lassosum we used the software of the same name (version 0.4.5). For LDpred-funct we used the software of the same name (version 1.0.0). For LDpred2 we used bigsnpr (version 1.7.0). For AnnoPred, we used the software of the same name (version 1.0). Step-by-step scripts for repeating our analyses are provided in Supplementary Note 1.

For manuscripts utilizing custom algorithms or software that are central to the research but not yet described in published literature, software must be made available to editors and reviewers. We strongly encourage code deposition in a community repository (e.g. GitHub). See the Nature Research guidelines for submitting code & software for further information.

Data

Policy information about availability of data

All manuscripts must include a data availability statement. This statement should provide the following information, where applicable:

- Accession codes, unique identifiers, or web links for publicly available datasets
- A list of figures that have associated raw data
- A description of any restrictions on data availability

Individual-level UK Biobank data can be applied for from www.ukbiobank.ac.uk. Neale Lab summary statistics can be downloaded from www.nealelab.is/uk-biobank.

Summary statistics for the eight additional diseases can be downloaded from the websites of the corresponding studies: asthma (www.ebi.ac.uk/gwas/studies/GCST006862), atrial fibrillation (www.ebi.ac.uk/gwas/studies/GCST004296), breast cancer (bcac.ccge.medschl.cam.ac.uk/bcacdata/oncoarray), inflammatory bowel disease (www.ibdgenetics.org/downloads.html), prostate cancer (http://practical.icr.ac.uk/blog/?page_id=8164), rheumatoid arthritis (plaza.umin.ac.jp/~yokada/datasource/software.htm), schizophrenia (www.med.unc.edu/pgc/download-results/) and type 2 diabetes (diagram-consortium.org/downloads.html).

Field-specific reporting

Please select the one below that is the best fit for your research. If you are not sure, read the appropriate sections before making your selection.

[x] Life sciences [ ] Behavioural & social sciences [ ] Ecological, evolutionary & environmental sciences

For a reference copy of the document with all sections, see nature.com/documents/nr-reporting-summary-flat.pdf

Life sciences study design

All studies must disclose on these points even when the disclosure is negative.

Sample size

We first analysed data for 220k individuals for each of 14 phenotypes. This was the number of individuals that remained after strict quality control of the UK Biobank data (we considered it appropriate to perform strict quality control because the performance of a prediction model can be sensitive to genotyping errors, population stratification and familial relatedness). The aim of our work is to show that improving the heritability model improves prediction, and it is clear from the results that 220k samples was sufficient to achieve this aim (for example, Figure 2 shows that the precision of results is sufficiently high to demonstrate the impact of improving the heritability model, while for the best-performing method, the improvement is over 8 standard deviations). We subsequently extended the analysis to 225 phenotypes, with average sample size 285k, finding that the results from the additional 211 phenotypes replicate those from the first 14.

Data exclusions

We provide a full description of our quality control of UK Biobank data in Methods. In total, UK Biobank provides data for 487k individuals. First we identified 419k individuals who were both recorded and inferred through principal component analysis to be white British, and who had values recorded for the covariates age, sex and Townsend Deprivation Index. Then for each of the 14 phenotypes in turn, we filtered the individuals with values for that phenotype so that no pair remained with allelic correlation >0.0325 (that expected for second cousins). Depending on phenotype, 220,399-253,314 individuals remained (in total, 392,214 unique). From the individuals that remained for each phenotype, we randomly picked 200,000 and 20,000 individuals to use for training and testing prediction models. When analysing all 225 phenotypes, we used summary statistics computed by the Neale Lab (www.nealelab.is/uk-biobank). In total, they provide results for 4,203 phenotypes. We downloaded results for the 283 phenotypes that were computed using both sexes (rather than only males or only females) and that had estimated SNP heritability >0.05. We primarily focused on the 225 phenotypes for which it was possible to construct a prediction model with R2 >0.01. We made this choice because it is difficult to reliably compare the performance of prediction tools using models with low and often non-significant R2. However, we nonetheless confirmed the patterns we saw across the 225 phenotypes with R2 >0.01 were replicated across the 58 phenotypes with R2 <0.01.

Replication

Our paper demonstrates that improving the heritability model leads to substantially improved prediction accuracy for a wide range of phenotypes and prediction tools. We first considered 14 phenotypes and four prediction tools that use individual-level data. We showed that improving the prediction model resulted in better prediction accuracy for all 14 phenotypes and all four prediction tools (i.e., for each of 14x4=56 analyses). We then considered 225 phenotypes and four prediction tools that use summary statistics. We showed that improving the prediction model resulted in better prediction accuracy for 223-225 phenotypes (in total, for 898 out of 225x4=900 analyses). Therefore, our result was replicated many times (i.e., for 100s of different phenotypes and for eight different prediction tools).

Randomization

When dividing samples into training and test datasets, this was done at random.

Blinding

Not applicable, as we did not collect the data ourself (instead, it came from the UK Biobank). Further, the phenotypes we considered are easy to measure (e.g., height, BMI, forced vital capacity), meaning there is limited scope for experiemental error (and even were there, there is no reason these would cause systematic bias in our results).

Reporting for specific materials, systems and methods

We require information from authors about some types of materials, experimental systems and methods used in many studies. Here, indicate whether each material, system or method listed is relevant to your study. If you are not sure if a list item applies to your research, read the appropriate section before selecting a response.

Materials & experimental systems

n/a	Involved in the study
[x]	[ ] Antibodies
[x]	[ ] Eukaryotic cell lines
[x]	[ ] Palaeontology and archaeology
[x]	[ ] Animals and other organisms
[ ]	[x] Human research participants
[x]	[ ] Clinical data
[x]	[ ] Dual use research of concern

Methods

n/a	Involved in the study
[x]	[ ] ChIP-seq
[x]	[ ] Flow cytometry
[x]	[ ] MRI-based neuroimaging

Human research participants

Policy information about studies involving human research participants

Population characteristics	We used data collected by UK Biobank between 2006 and 2010. In total, they recruited about 500,000 people from across the United Kingdom. For the 392,214 individuals we used (across 14 phenotypes), the ages ranged from 39 to 73 (interquartile range 51-63, mean 57, median 58), 212,094 (180,120) were female (male), and their Townsend Deprivation indexes ranged from -6.3 to 10.9 (interquartile range -3.7-0.1, mean -1.6, median -2.3)
Recruitment	UK Biobank recruited individuals by asking the (UK) National Health Service / Department of Health to randomly suggest people aged between 40 and 69; UK Biobank then sent these people invitations to attend an assessment centre. This continued until 500,000 people had agreed to take part. While UK Biobank sought to obtain a fully representative sample, those who accepted the invite tended to be more wealthy and healthy than average. However, these biases should not affect our analyses. This is because our analyses focus on relative differences (i.e., comparisons between different heritability models and different prediction tools), each of which should be impacted evenly by any biases in the sample collection.
Ethics oversight	The UK Biobank has approval from the North West Multi-centre Research Ethics Committee (MREC), which covers the United Kingdom. It also sought the approval in England and Wales from the Patient Information Advisory Group (PIAG) for gaining access to information that would allow it to invite people to participate. Further, the UK Biobank established a public Ethics and Governance Framework to set standards for the project, and to ensure that safeguards are in place for scientifically and ethically approved research. This framework was subject to a public consultation, and there was an independent Ethics and Governance Council established to ensure UK Biobank adheres to the framework. To access the data, we had to apply and gain approval from the UK Biobank.

Note that full information on the approval of the study protocol must also be provided in the manuscript.
