# Supplementary material for: Improved genetic prediction of complex traits from individual-level data or summary statistics
Source: Nat Commun. 2021 Jul 7;12:4192. doi: 10.1038/s41467-021-24485-y (PMC8263809; doi:10.1038/s41467-021-24485-y)
Supplement: Supplementary file 2 — Peer Review File [file 41467_2021_24485_MOESM2_ESM.pdf]

Editorial Note: This manuscript has been previously reviewed at another journal that is not operating a transparent peer review scheme. This document only contains reviewer comments and rebuttal letters for versions considered at Nature Communications.

REVIEWERS' COMMENTS

Reviewer #1 (Remarks to the Author):

I thank the authors for the clarification regarding which tools to use in practice, and highlighting the consistent performance of newly proposed methods. The authors have addressed my comments.

Reviewer #2 (Remarks to the Author):

The authors have revised the paper and addressed my major concerns, especially through the new Supplementary Note 4, with sufficient simulations regarding the R2/h2 point. I would like to state here that even though the authors find this concern of mine "niche", I was actually not questioning that the prediction performance could be improved by the new models. The new simulation finally shows that greater relative improvement for low-R2 traits is a property of the model itself (technically) instead of any particular genetic architecture of the selected low-h2 traits (biologically).

I do not have any further comments.

Comments to third reviews.

The reviewers' comments are in black, our responses are in red.

##

Reviewer #2 (Remarks to the Author):

The authors have revised the paper and addressed my major concerns, especially through the new Supplementary Note 4, with sufficient simulations regarding the R2/h2 point. I would like to state here that even though the authors find this concern of mine "niche", I was actually not questioning that the prediction performance could be improved by the new models. The new simulation finally shows that greater relative improvement for low-R2 traits is a property of the model itself (technically) instead of any particular genetic architecture of the selected low-h2 traits (biologically).

I do not have any further comments.

We disagree with the sentence "I was actually not questioning that the prediction performance could be improved by the new models". In the two previous rounds of review, the reviewer has repeatedly asserted that our new tools only improve performance for low R2/h2 phenotypes. For example, in their second review, the reviewer said "if improvement in prediction only matters for low-h2 and low-R2 traits when changing the heritability model, this should be clearly stated in abstract & conclusions". In other words, the reviewer previously told us that we should add that changing the heritability model only improves prediction for low R2/h2 phenotypes (despite us having shown that our new tools significantly improve performance for a wide range of phenotypes, regardless of factors such as R2 or h2).

At each review round, we have fully responded to the reviewer's comments. This has included performing all the analyses they requested, even when we considered them tangential or based on misunderstandings of our paper. By contrast, the reviewer has twice declined to respond to any of our comments (in both our first and second responses, we pointed out many errors in the reviewer's comments, yet in their both their second and third reviews, the reviewer refused either acknowledge or dispute any of our points).

##

Reviewer #3 (Remarks to the Author):

I thank the authors for the clarification regarding which tools to use in practice, and highlighting the consistent performance of newly proposed methods. The authors have addressed my comments.

We have no direct comments to Reviewer 3.

##

We are thoroughly disappointed with the treatment of our paper. It is at least some comfort that Nature Communications publishes the peer reviews and our responses, so that the reader can judge for themselves the quality of the review process and journal handling.
